# Draft Genome Sequence of a Dissimilatory U(VI)-Reducing Bacterium, Shewanella xiamenensis Strain DCB2-1, Isolated from Nitrate- and Radionuclide-Contaminated Groundwater in Russia

**DOI:** 10.1128/genomeA.00555-18

**Published:** 2018-06-21

**Authors:** Denis S. Grouzdev, Alexey V. Safonov, Tamara L. Babich, Tatiyana P. Tourova, Maria S. Krutkina, Tamara N. Nazina

**Affiliations:** a Institute of Bioengineering, Research Center of Biotechnology, Russian Academy of Sciences, Moscow, Russian Federation; b Frumkin Institute of Physical Chemistry and Electrochemistry, Russian Academy of Sciences, Moscow, Russian Federation; c V.I. Vernadsky Institute of Geochemistry and Analytical Chemistry of Russian Academy of Sciences, Moscow, Russian Federation; d Winogradsky Institute of Microbiology, Research Center of Biotechnology, Russian Academy of Sciences, Moscow, Russian Federation

## Abstract

Here, we describe the draft genome sequence of Shewanella xiamenensis strain DCB2-1, isolated from nitrate- and radionuclide-contaminated groundwater. This strain is able to reduce nitrate, Tc(VII), Cr(VI), Fe(III), and U(VI), and its genome sequence contains several gene sets encoding denitrification, resistance to heavy metals, and reduction of metals and metalloids.

## GENOME ANNOUNCEMENT

Bacteria of the Shewanella genus are metabolically versatile and are potentially capable of altering the solubility of a broad range of priority radionuclides, including uranium, other actinides, and fission products (1, 2). Members of this genus have often been revealed in radionuclide-contaminated environments (3, 4). Shewanella xiamenensis strain DCB2-1 (VKM В-3220) was isolated from a groundwater sample obtained near a suspended surface repository for radioactive waste in Russia. Groundwater was contaminated with strontium, uranium, nitrate, and sulfate ions. Strain DCB2-1 was isolated during the course of *in situ* trials of a groundwater bioremediation biotechnology for removal of nitrate ions and decrease in the radionuclide migration based on an injection of milk whey. Based on the 16S rRNA gene phylogeny, strain DCB2-1 is most closely related to Shewanella xiamenensis S4^T^ isolated from coastal sea sediment, which is capable of reducing nitrate, nitrite, selenite, fumarate, and ferric oxide under anaerobic conditions (5). Strain DCB2-1 grew optimally at 13°C, pH 7.0, and salinity at 10 g NaCl/liter, with these parameters being close to those at the sampling site. Under anaerobic conditions, the strain grew in media with acetate, lactate, and milk whey, using nitrate, pertechnetate [Tc(VII)], chromate [Cr(VI)], Fe(III) oxide, or uranium(VI) as electron acceptors. Analysis of the genome sequence for this new strain was therefore of great importance.

Genomic DNA was isolated from the biomass by a phenol-chloroform-based method, as described previously (6). The DNA was sonicated on a Covaris S2 device to an average fragment size of 250 bp. The libraries were constructed with NEBNext DNA library prep reagent set for Illumina, according to the protocol for the kit. The libraries were sequenced using an Illumina HiSeq 1500 platform with 230-bp read length. Finally, 12,080,351 reads were used for *de novo* assembly of the genome with SPAdes 3.10.1 (7). Identification of protein-coding sequences was performed using the NCBI Prokaryotic Genome Automatic Annotation Pipeline (PGAAP) (8). The draft assembly was annotated using the Rapid Annotations using Subsystems Technology (RAST) server (9).

The draft genome sequence of strain DCB2-1 consisted of 100 contigs representing an overall 4,736,625 bp, with an average G+C content of 46.3%. The genome contained 4,216 genes, of which 4,038 were protein-coding genes, 75 coded for tRNAs, and 4 belonged to noncoding RNAs (ncRNAs). In the genome, the genes involved in central carbon metabolism included a set of genes encoding glycolysis/gluconeogenesis, the Entner-Doudoroff pathway, the pentose phosphate pathway, and the tricarboxylic acid (TCA) cycle. Numerous genes responsible for the degradation of aromatic compounds, amino acids, and sugars, denitrification, heavy metal tolerance, and detoxification were identified in the genome, including those encoding cobalt-zinc-cadmium, chromium, arsenic, and fluoroquinolone resistance, siderophore assembly, uptake of selenate and selenite, and ferric and arsenate reductases. The draft genome sequence of S. xiamenensis strain DCB2-1 will improve our understanding of its potential for bioremediation of radionuclide-contaminated environments and expand our knowledge of the physiology of the Shewanella genus.

### Accession number(s).

This draft genome sequence has been deposited at DDBJ/ENA/GenBank under the accession number QFLG00000000. The version described in this paper is version QFLG01000000.
